# Clinical Applicability of Tissue Polypeptide Antigen and CA-125 in Gynecological Malignancies

**DOI:** 10.3390/biomedicines11112960

**Published:** 2023-11-02

**Authors:** Lars Schröder, Christian M. Domroese, Alexander B. A. Rupp, Kathrin M. E. Gihr, Christoph Niederau, Michael R. Mallmann, Stefan Holdenrieder

**Affiliations:** 1 Department of Obstetrics and Gynecology, Medical Faculty, University Hospital Cologne, University of Cologne, 50937 Cologne, Germany; schroeder.l@ketteler-krankenhaus.de; 2 Department of Obstetrics and Gynecology, Ketteler Hospital, 63071 Offenbach, Germany; 3 Institute of Clinical Chemistry and Clinical Pharmacology, University Hospital Bonn, 53127 Bonn, Germany; 4 Munich Biomarker Research Center, Institute of Laboratory Medicine, German Heart Center Munich, Technical University Munich, Lazarettstraße 36, 80636 Munich, Germany; 5 MVZ Labor Dortmund, 44147 Dortmund, Germany; 6 CEBIO GmbH—Center for Evaluation of Biomarkers, 81679 Munich, Germany

**Keywords:** ovarian cancer, uterine cancer, diagnosis, tumor marker, CA 125, TPA

## Abstract

Background: Nowadays there still is no sufficient screening tool for ovarian and uterine cancer. Objective: The current study aimed to investigate whether cancer antigen 125 (CA-125), tissue polypeptide antigen (TPA) or the combination of both markers are able to act as screening tools for ovarian or uterine cancer. Methods: A total of 275 blood samples from different cohorts (ovarian cancer, uterine cancer, benign control group) were prospectively drawn and analyzed. Results: Established biomarkers TPA and CA-125 showed elevated serum concentrations in patients with malignant tumors as compared to healthy women and women with benign diseases. In ROC curve analyses, both biomarkers were well able to discriminate between malignant and healthy, benign or overall non-malignant cases in the whole sample, with AUCs of 0.842 and above. While TPA was the best diagnostic marker in patients with uterine cancer, CA 125 was the best in patients with ovarian cancer. Conclusions: TPA and CA-125 both showed promising results for the detection of gynecologic malignancies. The combination of CA-125 and TPA did not improve sensitivity in comparison to single markers.

## 1. Introduction

Epithelial ovarian cancer (EOC) is the second most frequent malignancy of the female genital tract and the most lethal gynecologic cancer in developed countries [1]. While localized stage I tumors possess high 5-year survival rates of above 90%, the survival rates drop drastically in advanced stages [2]. Although some types of ovarian cancer are typically diagnosed in relatively early stages, the most common type, i.e., EOC, often has already progressed into advanced stages at the time of diagnosis due to the lack of distinct early symptoms. Hence, there is a need for criteria for the early diagnosis of ovarian cancer. Currently, diagnostic guidelines basically rely on thorough anamnesis and ultrasound, as well as other radiological methods, and the use of blood-based tumor markers such as cancer antigen 125 (CA-125) [3,4]. Further, combinations of CA-125 with other blood-based markers such as tissue polypeptide antigen (TPA) may be beneficial not only in diagnosis but also in treatment monitoring of ovarian cancer [5,6]. However, although sensitivities and specificities are often reported to be in a satisfying range, there is still no ideal algorithm in a manner that would allow for comprehensive screening programs [7]. In addition, some tumor markers including CA-125 are well known to be highly influenced not only by possible ovarian malignancies but also in the course of physiological changes such as menses or menopause [8].

Unlike ovarian cancer, uterine malignancies often show symptoms in an early stage, above all, abnormal vaginal discharge or bleeding. Hence, they are often diagnosed in a localized stage. However, both the incidence and mortality rates of uterine cancer are generally increasing. Additionally, no routine screening method is currently applied to detect uterine malignancies early on [9,10]. However, a multitude of biomarkers have been investigated as possible prognostic or predictive parameters in malignant uterine tumors, including RCAS1 [11], HE4 [12] and the P2X7 receptor [13]. For instance, a recent meta-analysis showed that over 250 proteins have been associated with different aspects of endometrial cancer such as overall survival, myometrial invasion or lymph node status [14]. Comparable to ovarian cancer, CA-125 has also been studied extensively in uterine tumors and has been used for the differentiation of endometrial cancer and abnormal uterine bleeding or for assessment before surgical treatment, for instance [15,16,17]. However, heterogeneity in studies concerning CA-125 in endometrial cancer is high and its diagnostic or prognostic value is not entirely clear at the moment [14]. Likewise, results for other uterine malignancies like sarcomas are not consistent across studies [18,19,20].

In this study, concentrations of the biomarkers TPA and CA-125 were determined in blood samples of 275 women with ovarian cancer, uterine cancer, ovarian cysts, other benign diseases or without any diagnosed severe disease. A regression model was applied to assess the applicability of both biomarkers as a potential screening tool for ovarian and uterine cancer. ROC curves were analyzed for TPA, CA-125 and the combination of TPA and CA-125, in order to assess whether TPA is comparable or additive to CA-125 in terms of diagnostic power.

## 2. Materials and Methods

The study was conducted in accordance with the Declaration of Helsinki and approved by the Institutional Review Board (Nr. 260/13) of the Medical Faculty of the Rheinische Friedrich-Wilhelms-University in Bonn, Germany. Informed consent was obtained from all subjects involved in the study.

### 2.1. Participants and Samples

Blood samples were drawn between 2009 and 2015 at the Department of Obstetrics and Gynecology of the University Hospital Bonn (Germany) during routine sample collection for the Biobank of the Institute of Clinical Chemistry and Clinical Pharmacology (University Hospital Bonn). Samples of patients suffering from malignant gynecologic disease were constantly collected before the start of therapy after cubital venipuncture in gel serum tubes (Sarstedt, Nurmbrecht, Germany). After drawing, samples were transferred to the central lab, centrifuged, and serum was aliquoted and stored at −80 °C in the Biobank until needed for measurements. Patients with a diagnosis of ovarian cancer or uterine cancer were used as the malignancy group, while patients with non-malignant diagnoses of the ovaries, fallopian tubes or uterus were defined as the benign group. Exclusion criteria were chronic kidney disease, diabetes, HIV and hepatitis infection, as well as other current or former non-gynecologic malignancies. The group of healthy controls comprised female individuals without known gynecologic or malignant diagnoses.

### 2.2. Assays

Concentrations of CA-125 and TPA were determined with a chemoluminescence immunosorbent assay on a Diasorin LIAISON XL system (Diasorin S.p.A., Saluggia, Italy) at the MVZ Laboratory Dr. Niederau, Dortmund, Germany.

### 2.3. Statistics

Statistical analyses were performed with IBM SPSS Statistics Version 23. The significance level was set to α = 0.05 for all analyses. Differences in age and biomarker concentrations between two groups were tested with Mann–Whitney U tests and with Kruskal–Wallis tests between more than two groups. Correlations between measured biomarkers were tested with Spearman correlations. The Bonferroni method was used for the correction of multiple testing. Diagnostic abilities of CA-125 and TPA were assessed using receiver operating characteristics (ROCs). Logistic regression models with log10 concentrations of TPA and CA-125 as predictor variables were used to investigate predictive power of TPA and CA-125 in the prediction of malignancies.

## 3. Results

Of the 275 women included in this study, 58 were healthy controls, 124 had benign diseases, 70 suffered from ovarian malignancies and 23 were diagnosed with uterine cancer. Benign entities were further subdivided into ovarian cysts (N = 23), other benign conditions of the ovaries or salpinges (N = 53) and benign uterine diseases (N = 48). Malignant ovarian cases consisted of primary (N = 43) and recurring tumors (N = 27) before the start of therapy, respectively. Of the malignant ovarian tumors, five (7%) were classified as FIGO stage I, two (3%) as FIGO stage II, 41 (59%) as FIGO stage III and three (4%) as FIGO stage IV. For the 19 ovarian malignancies (27%), no data concerning FIGO classification was available. Further, uterine malignancies consisted of 11 cases (48%) of endometrial cancer, 7 cases (30%) with different types of uterine sarcomas and 5 patients (22%) with uterine malignancies of mixed histologic classification. No FIGO or TNM classifications were available for uterine malignancies.

As given in Table 1, the groups differed significantly (*p* < 0.001) in age, with women suffering from ovarian and uterine malignancies having the highest median ages of 62 and 63 years, respectively, and healthy women possessing a median age of only 43 years.

**Figure 1:**
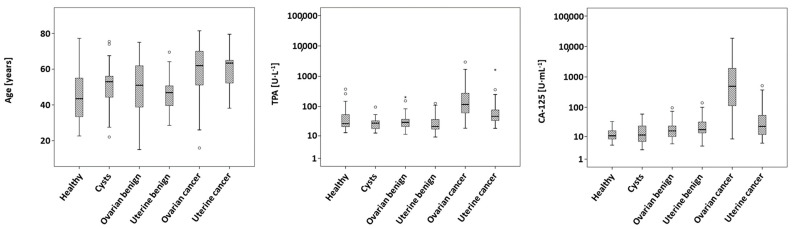
Boxplots of age, concentrations of TPA and concentrations of CA-125 stratified according to diagnosis.

Further, measured levels of TPA (*p* < 0.001) and CA-125 (*p* < 0.001) show significant differences between the different entities, which remained significant after Bonferroni correction. Ovarian cancer exhibited the highest median concentrations both in TPA and in CA-125, while uterine malignancies showed only the second-highest concentrations. Measured concentrations of healthy controls, cysts and other benign ovarian or uterine diseases yielded consistently lower values. Boxplots of age, TPA and CA-125 are shown in Figure 1.

Spearman correlations between TPA and CA-125 showed significantly positive correlations when all participants (ρ = 0.470, *p* < 0.001) or only malignant cases (ρ = 0.693, *p* < 0.001) were considered, but not when only benign cases (ρ = 0.069, *p* = 0.445) or only healthy controls (ρ = −0.107, *p* = 0.425) were taken into account (Table 2). As given in Table 3, both TPA and CA-125 were able to significantly distinguish malignant cases from healthy controls, benign cases or all non-malignant participants, with AUCs ranging between 0.839 and 0.902. Significant discrimination between healthy controls and benign cases was only achieved by CA-125 (AUC: 0.674, *p* < 0.001), but not by TPA (AUC: 0.437, *p* = 0.173). In the calculation of sensitivities at defined specificities of 90% (Sens90) and 95% (Sens95), the highest values were obtained for the differentiation between healthy controls and malignancies with CA-125 (80.6%/75.3%) and for the differentiation between all non-malignant and malignant cases with CA-125 (71.0%/64.5%).

Restricting ROC curve analyses to patients with uterine diagnoses and healthy controls gave the results presented in Table 4. Both biomarkers yielded somewhat lower AUCs compared to results from all participants in the range of 0.568–0.805. TPA as well as CA-125 (*p* < 0.001) were well able to discriminate between malignant cases and healthy controls or all non-malignant cases, with AUCs between 0.664 and 0.764. While discrimination between healthy women and those with benign uterine diagnoses was achieved by both biomarkers, only TPA (*p* < 0.001), but not CA-125 (*p* = 0.357), gave a significant AUC in the differentiation between benign and malignant cases. Values of Sens90 and Sens95 generally were substantially lower than in the ROC curve analyses of the whole study sample, with ranges between 25.9 and 56.5% for Sens90 and 13.0 and 39.1% for Sens95.

**Figure 2:**
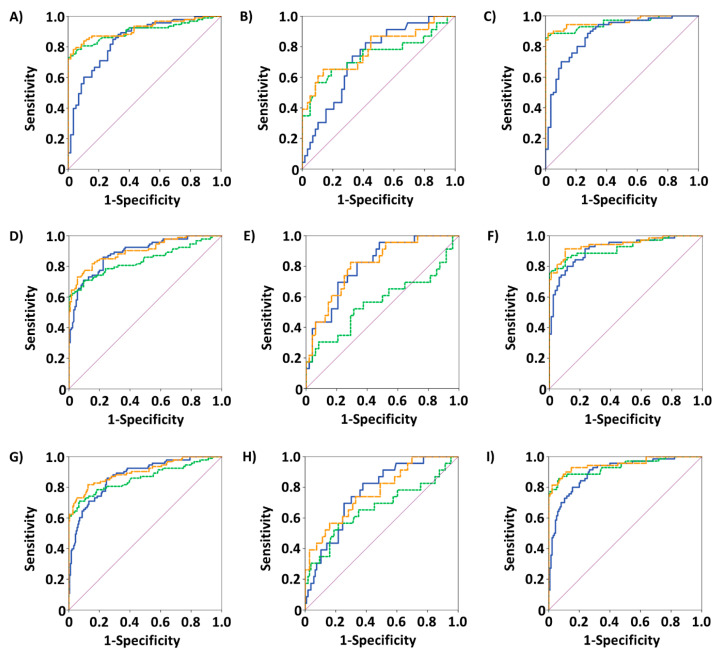
ROC curves from differentiation between healthy and malignant (**A**–**C**), benign and malignant (**D**–**F**) or non-malignant and malignant (**G**–**I**) cases. First column: ROC curves of all respective participants; middle column: ROC curves of respective uterine cases; last column: ROC curves of respective ovarian cases. Blue continuous curves: TPA; green dotted curves: CA-125; orange dashed curves: based on logistic regression models with log_10_ concentrations of TPA and CA-125.

Analyses of ROC curves comprising data from healthy individuals and patients with a benign or malignant ovarian diagnosis are summarized in Table 5. A graphical representation is depicted in Figure 2. Again, both TPA and CA-125 were able to distinguish malignant cases from healthy controls, ovarian cysts, all benign or all non-malignant cases (all *p* < 0.001), resulting in AUCs of 0.879–0.954, as well as Sens90 of 65.7–88.6% and Sens95 of 48.6–88.6%. Furthermore, a significant discrimination (*p* < 0.001) between primary and recurring ovarian malignancies was achieved by both biomarkers (AUCs: 0.762 for TPA, 0.802 for CA-125).

Results from binomial logistic regression models with log10 concentrations of TPA (in U/mL) and CA-125 (in U/L) as predictor variables and the malignant diagnosis as the dependent variable are shown in Table 6. Odds ratios (ORs) greater than 1—indicating a higher risk of suffering from malignant tumors with increasing serum concentrations—were obtained for TPA and CA-125, respectively, in all calculated models. All contributions of log10(TPA) and log10(CA-125) were significant with the exception of log10(TPA) in the model comprising healthy women and ovarian malignancies (*p* = 0.052) and log10(CA-125) in the prediction of malignancies from the group of benign and malignant uterine cases (*p* = 0.438). In general, higher odds ratios were found for log10(CA-125) in models with healthy and malignant cases. In the comparison of all healthy and all malignant cases, a 10-fold increase in the CA-125 concentration was associated with an OR of 80.4 (*p* < 0.001), whereas a 10-fold increase in TPA resulted in an OR of 8.26 (*p* = 0.008). When healthy controls and uterine cancer were taken into account, ORs of 42.6 (log10(CA-125)) and 7.53 (log10(TPA)) were calculated, while the respective model with ovarian cancer yielded ORs of 319 (log10(CA-125)) and 9.24 (log10(TPA), *p* = 0.052). Regression models with benign and malignant cases showed higher ORs for log10(TPA), with the largest difference in benign and malignant uterine diagnoses (54.9 vs. 1.76) and the smallest differences in ovarian malignant and benign cases (22.5 vs. 17.0). Comparing all non-malignant with all malignant cases resulted in ORs of 17.7 (log10(TPA)) and 13.0 (log10(CA-125)). Respective ORs in the model with healthy controls and uterine cases were 13.3 and 7.19. In the model with ovarian cases, ORs of 10.8 (log10(TPA)) and 39.6 (log10(CA-125)) were found.

Calculation of ROC curves from logistic regression models for the detection of malignant cases gave the results presented in Table 7. All AUCs were highly significant (*p* < 0.001) and were highest (0.954, 0.940 and 0.943) in the respective subgroups with ovarian cases. These models also yielded the highest Sens90 (81.4–88.6%) and Sens95 (77.1–88.6%). The AUC resulting from the logistic regression of non-malignant vs. malignant uterine cases gave the lowest value of 0.755, as well as the lowest Sens90 (43.5%) and Sens95 (34.8%).

## 4. Discussion

Ovarian and uterine cancer entities annually account for over 200,000 deaths worldwide [1]. While ovarian cancer is often diagnosed in late stages due to the lack of early symptoms, endometrial cancer is accompanied by vaginal bleeding or other symptoms. Still, such symptoms may remain unrecognized or misinterpreted by patients as a physiologic phenomenon. Currently, the use of biomarkers as a screening tool for ovarian or endometrial cancer is not recommended, above all, due to a lack of mortality reduction [21,22,23]. The combination of CA-125 and transvaginal ultrasound may be used for early detection of ovarian cancer, but it is not recommended as a screening tool for the general population [24]. Therefore, establishing a reliable and easily accessible set of clinical tumor markers as a screening tool for the detection of early stage ovarian and uterine cancer is highly desirable. In this study, the concentration of the biomarkers TPA and CA-125 were measured in serum samples of women with malignant ovarian and uterine tumors, a variety of benign diseases including ovarian cysts and of women without known severe diseases.

Malignant cases consisted mostly of serous carcinoma (61%) and were mainly in FIGO stage III (60%), which can be considered representative of ovarian malignant tumors [2,25,26]. Moreover, the histologic distribution of uterine malignancies is in line with epidemiologic data [27].

As expected, and in accordance with the literature data [5,28,29,30], the established biomarkers TPA and CA-125 showed elevated concentrations in malignant tumors as compared to healthy women and benign diseases, with median values of uterine cancer being moderately elevated and those of ovarian cancer being strongly elevated. In accordance with the elevated levels of both CA-125 and TPA in malignant subgroups, concentrations of CA-125 and TPA were correlated in the whole sample as well as in malignant cases, but not in benign cases or healthy participants. It is known that CA-125, but not TPA is elevated in benign diseases, which explains the lack of correlation in the benign group. Interestingly, no significant differences in biomarker concentrations were found between serous and non-serous ovarian malignancies. This finding is even more remarkable as CA-125 is explicitly stated by the European Group on Tumor Markers as a suitable biomarker in combination with other diagnostics for detection, monitoring and prognosis of serous ovarian carcinomas due to its relatively high concentrations [3]. In the case of CA-125, it is well known that serum levels decrease with age and in postmenopausal women [31]. However, age as well as FIGO stage distribution did not differ significantly between serous and non-serous malignancies. A non-significant tendency towards higher age, higher TPA and higher CA-125 concentrations was present in patients with serous ovarian cancer, which may indicate that an underlying difference can be detected in a larger sample. A definite explanation cannot be given at the moment, since—as stated before—no difference was present in terms of FIGO stages. Due to the lack of other basic characterization data, there may, however, exist other hidden confounding variables. For instance, hormone replacement therapy, menopausal status, a history of hysterectomy, smoking, caffeine consumption and ethnicity have been shown to influence concentrations of CA-125 in healthy women [32,33].

Concentrations of TPA and CA-125 were significantly higher in primary than in recurrent tumors, while FIGO stages and age did not differ between both subgroups. However, it may be hypothesized that the absolute tumor mass is higher on average in primary tumors, as recurrent tumors tend to be found in the course of follow-up care. Thus, recurrent tumors may well have spread to peritoneal tissue outside the pelvis or into retroperitoneal lymph nodes, which categorizes as FIGO stage III, even if the total mass is relatively low. This will hold true even more if the primary tumor was already not limited to the ovaries. Therefore, there may on average be less tissue present to produce or induce the production of the respective biomarkers, resulting in the differences shown.

In ROC curve analyses, both biomarkers were well able to discriminate between malignant and healthy, benign or overall non-malignant cases in the whole sample, with AUCs of 0.842 and above. Furthermore, sensitivities at specificities of 90% (Sens90) and 95% (Sens95) were higher in the case of CA-125, reaching values of up to 80.6% and 75.3% in the differentiation between healthy controls and malignant diagnoses. The differentiation between healthy and benign cases yielded lower—and in the case of TPA, non-significant—AUCs, which is a result of the smaller differences in concentrations and overlapping concentration ranges of healthy and benign participants. Analyses of ROC curves concerning uterine cases and healthy controls revealed considerably lower AUCs of 0.568 to 0.805 and likewise lower values of Sens90 and Sens95. As mentioned before, biomarker concentrations in uterine cancer were elevated compared to healthy and benign cases, yet to a lesser degree as ovarian cancer cases. Therefore, discrimination between malignant and any non-malignant cases was expected to be more difficult, as represented by lower AUCs and sensitivities. Concordant with this implication, ROC curve analyses comprising ovarian cancer cases and healthy controls resulted in even higher AUCs than in the analyses of the whole sample. Moreover, while differentiation between serous and non-serous malignancies was not significant in ROC curve analyses, TPA and CA-125 were able to distinguish primary from recurring malignant ovarian tumors to a similar extent, albeit with considerably smaller Sens90 and Sens95 of well below 50%.

Logistic regression models for the prediction of malignant tumors with log10(CA-125) and log10(TPA) as continuous predictor variables consistently yielded odds ratios above 1. As expected, log10(TPA) showed higher ORs than log10(CA-125) when only benign and malignant diagnoses were included in the models. This represents the fact that a variety of benign diseases lead to elevated concentrations of CA-125, but not TPA, thereby diminishing differences in CA-125 and TPA between benign and malignant cases [24]. Accordingly, contributions of log10(CA-125) were higher than those of log10(TPA) when benign cases were omitted. In general, ORs of log10(CA-125) were highest in models comprising only ovarian cases with or without healthy controls. As mentioned above, CA-125 may be used together with transvaginal ultrasound for the detection of malignant ovarian tumors. Therefore, high ORs in these models reflect the known high sensitivity of CA-125 in the diagnosis of ovarian cancer. Altogether, all logistic regressions provided highly significant models for the prediction of malignant gynecologic diagnoses with a combination of CA-125 and TPA.

Further evaluation of the results from the logistic regression was performed by calculating ROC curves from regression models. These ROC curves yielded similar or even higher AUCs and sensitivities than the respective ROC curves based on the single biomarkers. Again, ovarian cases and healthy controls showed the most promising results, i.e., the highest AUCs, Sens90 and Sens95. These results correspond to results from regression analyses as well as from ROC curves of the single biomarkers. The combination of TPA and CA-125 therefore showed a good diagnostic ability in the detection of malignant tumors not only concerning ovarian cancer but also in the whole study cohort consisting of healthy women, as well as ovarian and uterine benign and malignant cases.

In modern treatment of malignant diseases including gynecologic types of cancer, an interdisciplinary approach has become the state-of-the-art treatment, by which not only a high quality of care is provided but also adherence to current guidelines in diagnosis, treatment and follow-up care is ensured [34,35]. In this respect, new and easily accessible biomarkers can deliver more information to interdisciplinary teams by assisting in clinical decision making. However, the early detection of gynecologic malignancies remains a challenge. For instance, although CA-125 is seen as one of the most promising biomarkers in epithelial ovarian cancers, 20% of tumors are missed in initial screenings [36,37,38]. The combination of promising biomarkers is an auspicious strategy to achieve higher diagnostic precision and reliability with respect to clinical applicability. In the present study, single markers TPA in patients with uterine cancer and CA 125 in patients with ovarian cancers, as well as the combination of both markers, yielded high sensitivities and specificities, hence providing a starting point for further testing in larger cohorts. Likewise, Lv et al. also reported CA 125 and TPA as being the best diagnostic biomarker combination [6].

Of course, limitations of this study have to be considered when interpreting the results presented here. First, only few clinical characteristics were collected. Therefore, possible confounders such as smoking, pre- and postmenopausal status or body mass index could not be evaluated. No serial samples during therapy or follow-up were collected, since this study was designed as a proof of principle for the combination of CA-125 and TPA in gynecologic malignancies. Thereby, no information concerning changes in CA-125 and TPA in the course of treatment is available. However, such information would of course have to be collected in further studies before the combination of CA-125 and TPA can be applied in clinical decision making. The number of ovarian cancer patients in early stages (FIGO I/II) was very limited. This is an important limitation as it restricts the validity of the results concerning the early detection of ovarian cancer with TPA and CA-125. However, most ovarian malignancies—particularly serous carcinoma—are diagnosed in an advanced stage. Accordingly, the numbers and stage distribution of the cohort investigated reflect the set of patients who presented at the University Hospital Bonn—mainly in Figo Stage III. Additionally, no information concerning tumor staging was available for uterine tumors. Thus, no stratification into early and late stages could be performed for this group, making transferability of the results from the whole study cohort or subgroups comprising uterine cancer to the general population difficult. Moreover, it must be taken into account that different types of tumors were present, for instance, serous and non-serous ovarian tumors or carcinomas and sarcomas in the uterine group. However, the main focus of this study was to assess the clinical applicability of CA-125 and TPA for the general detection of gynecologic malignancies. Ideally, such an applicability should be employable independent of the mentioned characteristics, especially independent of the tumor type. Nevertheless, it has to be emphasized that the cohort reflects the patients treated in a University Hospital setting. Sample collection, preanalytical handling and storage were carried out in a well standardized manner, lab analyses were conducted highly quality-controlled on an automatized system and statistics were deployed independently from sample collection and lab analyses.

## 5. Conclusions

In summary, TPA and CA-125 both showed promising results for the detection of gynecologic malignancies as single biomarkers and also in combination. Despite the drawbacks mentioned above, the diagnostic abilities of TPA and CA-125 should be further evaluated in additional prospective studies, in which more clinical characteristics can be collected.

## Figures and Tables

**Table 1:** Characterization of the study cohort.

Parameter	Age ^1^	TPA ^2^	CA-125 ^3^
Healthy controls (N = 58)	43 (22)	26.8 (32.8)	10.7 (7.8)
Ovarian cysts (N = 23)	53 (13)	27.8 (17.2)	11.4 (19.3)
Other ovarian benign (N = 53)	51 (26)	29.6 (20.1)	15.9 (14.1)
Uterine benign (N = 48)	47 (11)	21.4 (20.1)	17.6 (19.2)
Ovarian cancer (N = 70)	62 (19)	117.8 (221.8)	495.0 (1864.5)
Uterine cancer (N = 23)	63 (13)	47.4 (48.4)	22.8 (45.2)
***p*** -value *	**<0.001**	**<0.001**	**<0.001**

Data are given as medians (interquartile range). * Results from Kruskal–Wallis tests. ^1^ Years; ^2^ data given in U∙L^−1^; ^3^ data given in U∙mL^−1^.

**Table 2:** Spearman correlations between TPA and CA-125 in different subgroups.

Subgroup	N	Correlation Coefficient	*p*-Value
All participants	275	0.470	**<0.001**
All malignant cases	93	0.693	**<0.001**
All benign cases	124	0.069	0.445
Healthy controls	58	−0.107	0.425

**Table 3:** Data from ROC curve analysis comprising all participants.

Comparison	Parameter	AUC	95% CI	*p*-Value	Sens_90_	Sens_95_
healthy vs. malignant	TPA	0.842	0.777–0.907	**<0.001**	55.9	39.8
	CA-125	0.902	0.853–0.951	**<0.001**	80.6	75.3
healthy vs. benign	TPA	0.437	0.348–0.527	0.173	4.8	1.6
	CA-125	0.674	0.595–0.752	**<0.001**	32.7	23.8
all benign vs. malignant	TPA	0.886	0.842–0.930	**<0.001**	71.0	58.1
	CA-125	0.839	0.780–0.898	**<0.001**	69.9	63.4
all non-malignant vs. malignant	TPA	0.872	0.829–0.916	**<0.001**	65.6	51.6
	CA-125	0.859	0.805–0.913	**<0.001**	71.0	64.5

Underlined diagnoses were used as classifiers. AUC = area under the curve; CI = confidence interval; Sens_90_ = percent sensitivity at 90% specificity; Sens_95_ = percent sensitivity at 95% specificity.

**Table 4:** Data from ROC curve analysis comprising healthy controls, uterine benign and uterine malignant cases.

Comparison	Parameter	AUC	95% CI	*p*-Value	Sens_90_	Sens_95_
healthy vs. malignant	TPA	0.729	0.614–0.844	**0.001**	26.1	13.0
	CA-125	0.744	0.605–0.883	**0.001**	56.5	34.8
healthy vs. benign	TPA ^a^	0.620	0.512–0.728	**0.034**	25.9	20.7
	CA-125	0.753	0.658–0.848	**<0.001**	37.5	29.2
benign vs. malignant	TPA	0.805	0.702–0.908	**<0.001**	43.5	39.1
	CA-125	0.568	0.410–0.726	0.357	30.4	21.7
all non-malignant vs. malignant	TPA	0.764	0.667–0.860	**<0.001**	30.4	17.4
	CA-125	0.664	0.524–0.805	**0.014**	34.8	30.4

Underlined diagnoses were used as classifiers. ^a^ Healthy status instead of benign status was used as classifier. AUC = area under the curve; CI = confidence interval; Sens_90_ = percent sensitivity at 90% specificity; Sens_95_ = percent sensitivity at 95% specificity.

**Table 5:** Data from ROC curve analysis comprising healthy controls and ovarian cysts, as well as other ovarian benign and ovarian malignant cases.

Comparison	Parameter	AUC	95% CI	*p*-Value	Sens_90_	Sens_95_
healthy vs. malignant	TPA	0.879	0.819–0.939	**<0.001**	65.7	48.6
	CA-125	0.954	0.918–0.989	**<0.001**	88.6	88.6
healthy vs. benign	TPA	0.473	0.373–0.573	0.591	5.2	2.6
	CA-125	0.625	0.531–0.718	**0.013**	29.7	19.5
cysts vs. malignant	TPA	0.937	0.888–0.986	**<0.001**	84.3	80.0
	CA-125	0.934	0.887–0.980	**<0.001**	84.3	80.0
all benign vs. malignant	TPA	0.914	0.869–0.960	**<0.001**	74.3	64.3
	CA-125	0.923	0.877–0.969	**<0.001**	80.0	78.6
all non-malignant vs. malignant	TPA	0.899	0.855–0.944	**<0.001**	70.0	58.6
	CA-125	0.936	0.896–0.976	**<0.001**	87.1	78.6
primary vs. recurring malignant	TPA	0.762	0.648–0.875	**<0.001**	32.6	31.0
	CA-125	0.802	0.690–0.915	**<0.001**	48.5	7.0

Underlined diagnoses were used as classifiers. AUC = area under the curve; CI = confidence interval; Sens_90_ = percent sensitivity at 90% specificity; Sens_95_ = percent sensitivity at 95% specificity.

**Table 6:** Results from binomial logistic regression analyses with malignant diagnosis as dependent variable and log10(TPA) and log10(CA-125) as continuous predictor variables.

Cohort	Parameter	Odds Ratio	95%-CI	*p*-Value
Healthy vs. malignant				
All participants	log_10_(TPA)	8.257	1.733–39.332	**0.008**
	log_10_(CA-125)	80.402	11.207–576.833	**<0.001**
Uterine cases	log_10_(TPA)	7.531	1.368–41.445	**0.020**
	log_10_(CA-125)	42.630	3.920–463.650	**0.002**
Ovarian cases	log_10_(TPA)	9.240	0.979–87.215	0.052
	log_10_(CA-125)	319.390	19.564–5214.299	**<0.001**
Benign vs. malignant				
All participants	log_10_(TPA)	38.035	8.276–174.801	**<0.001**
	log_10_(CA-125)	6.758	2.772–16.479	**<0.001**
Uterine cases	log_10_(TPA)	54.858	5.176–581.391	**0.001**
	log_10_(CA-125)	1.756	0.424–7.280	0.438
Ovarian cases	log_10_(TPA)	22.512	2.489–203.644	**0.006**
	log_10_(CA-125)	17.041	4.511–64.378	**<0.001**
Non-malignant vs. malignant				
All participants	log_10_(TPA)	17.679	5.190–60.217	**<0.001**
	log_10_(CA-125)	12.985	5.346–31.540	**<0.001**
Uterine cases	log_10_(TPA)	13.285	2.903–60.794	**0.001**
	log_10_(CA-125)	7.194	1.849–27.988	**0.004**
Ovarian cases	log_10_(TPA)	10.813	1.855–63.048	**0.008**
	log_10_(CA-125)	39.647	10.502–149.675	**<0.001**

CI = confidence interval.

**Table 7:** Results from ROC analyses of logistic regression models comprising CA-125 and TPA concentrations as continuous or dichotomous variables.

Model	AUC	95% CI	*p*-Value	Sens_90_	Sens_95_
Healthy vs. malignant					
All participants	0.912	0.867–0.958	**<0.001**	79.6	76.3
Uterine cases	0.778	0.650–0.907	**<0.001**	47.8	39.1
Ovarian cases	0.954	0.919–0.990	**<0.001**	88.6	88.6
Benign vs. malignant					
All participants	0.897	0.854–0.940	**<0.001**	73.1	64.5
Uterine cases	0.805	0.699–0.911	**<0.001**	47.8	39.1
Ovarian cases	0.940	0.901–0.980	**<0.001**	81.4	77.1
Non-malignant vs. malignant					
All participants	0.891	0.847–0.935	**<0.001**	73.1	69.9
Healthy and uterine cases	0.755	0.648–0.863	**<0.001**	43.5	34.8
Healthy and ovarian cases	0.943	0.905–0.980	**<0.001**	85.7	80.0

AUC = area under the curve; CI = confidence interval; Sens_90_ = percent sensitivity at 90% specificity; Sens_95_ = percent sensitivity at 95% specificity.

## Data Availability

Raw data can be provided from the authors on request.
